# An analysis of COVID-19 mortality regarding all Austrian political districts

**DOI:** 10.1007/s00508-026-02728-6

**Published:** 2026-03-26

**Authors:** Stefanie Grötz, Martin Fieder, Harald Wilfing

**Affiliations:** Department of Evolutionary Anthropology, University of Vienna, Vienna, Austria

**Keywords:** Pandemic, SARS-CoV-2, Coronavirus, Case fatality ratio, Mortality ratio

## Abstract

**Background:**

Coronavirus disease 2019 (COVID-19), first identified in Wuhan in 2019, rapidly escalated into a global pandemic. Austria reported its initial cases in February 2020, resulting in millions of infections and thousands of deaths. This study investigates COVID-19 mortality across Austrian districts, analyzing factors such as age, sex, immigrant background, household size, education and vaccination status.

**Methods:**

We analyzed data from the Austrian National Public Health Institute, comprising 4,591,739 confirmed cases and 20,123 deaths as of July 2022. We used a linear mixed model to examine whether individuals died from COVID-19. Furthermore, we created three interaction models: (i) vaccination status and consecutive month, (ii) age and consecutive month and (iii) sex and consecutive month. Additional linear models examined household size, education, and citizenship at the district level.

**Results:**

Our findings highlight several determinants of COVID-19 mortality in Austria. Advanced age significantly increases mortality risk, while vaccination is strongly protective. Female sex is also associated with reduced mortality. District of residence influences the case fatality ratio, with notable geographic variation. Larger household sizes correlate with increased infection risk. Districts with higher proportions of residents with tertiary education exhibit lower infection and mortality ratio. Districts with a greater percentage of non-Austrian citizens have lower case fatality ratio, likely reflecting a younger population demography.

**Conclusion:**

Key determinants of COVID-19 mortality include age, vaccination status, and sex. Districts with larger households experience higher infection ratios, while those with more highly educated residents have lower infection and mortality ratios. Districts with more non-Austrian citizens show lower case fatality ratios, likely due to younger populations. Further research into district-level age structures is warranted to refine these models. These findings underscore the importance of vaccination, particularly among older adults, and the need for accessible public health education to reduce infection and mortality ratio, especially in less-educated populations; however, district age structure (proportion of older individuals) substantially influences these associations.

## Background

Coronavirus disease 2019 (COVID-19), an airborne respiratory illness caused by severe acute respiratory syndrome coronavirus type 2 (SARS-CoV-2), emerged in Wuhan in late 2019 and rapidly became a global pandemic. By May 2025, over 777 million cases and more than 7 million deaths had been reported worldwide [1].

Austria reported its first COVID-19 cases in February 2020 and its first deaths in March 2020. The initial wave was marked by outbreaks in Tyrolean ski resorts linked to winter tourism, prompting the government to impose a nationwide lockdown on 16 March 2020. Despite early success in mitigating the first wave, Austria experienced repeated surges in infections, particularly affecting vulnerable populations such as older individuals in long-term care facilities. This led to additional lockdowns, both nationwide and in some regions of the country [2]. This resulted in criticism of the authorities, who were accused of negligence regarding the disease [3]. The introduction of vaccines in late 2020 marked a turning point; however, it would still take a few months until a sufficient quantity of vaccines was available in Austria [4]. Districts experienced varying impacts from COVID-19 in terms of infection rates and case fatality rates, which were influenced by factors such as population demographics or socioeconomic factors. This highlights the need to understand the localized factors influencing COVID-19 outcomes.

### Existing literature and knowledge gaps

COVID-19 has affected the districts of a country differently [5]. Several studies have shown that older age is associated with a greater likelihood of dying from COVID-19 [6, 7]. Sex also plays a role. Male sex is associated with increased mortality [8]. Similarly, people from an immigrant background are more likely to die from COVID-19 [9]. Household size is another factor influencing COVID-19 transmission and mortality. The greater the number of people living in a household, the higher the transmission and mortality [6]. Education also makes a difference with respect to deaths. People with tertiary education are more likely to survive COVID-19 [10] and COVID-19 vaccination is another factor that plays an important role in preventing deaths [11]; however, localized studies that integrate these variables to assess district-level disparities remain limited, particularly in Austria.

This study seeks to address these gaps by analyzing the impact of district-level COVID-19 in Austria, with a focus on the infection ratio and case fatality ratio. Using a dataset from the Austrian National Public Health Institute and aggregated data, the study examines age, sex, vaccination status, and sociodemographic characteristics such as household size, education and citizenship. By identifying district-specific patterns, the study aims to inform policy makers on effective pandemic responses.

## Methods

### Study aim, design and setting

This study aimed to investigate the factors influencing COVID-19 infection and mortality across different districts in Austria. By analyzing district-level data, this study sought to understand the role of demographic and vaccination factors in mortality outcomes. Austria, a small country in Central Europe with a population of approximately 9.1 million in 2023 [12], is divided into 9 federal provinces, comprising 116 districts, including the 23 districts in the capital, Vienna [13]. The study utilized data provided by the Austrian National Public Health Institute through the COVID-19 data platform, which recorded COVID-19 infections, deaths, vaccinations, hospitalizations, and biomedical data during the pandemic [14].

### Data

The dataset used for this analysis included demographic information (age, sex, and district of residence), vaccination status, and COVID-19-related fatalities up to 2022-07-14. It did not contain any information about the variant of the virus, as this is not relevant for our analysis. In our research, we analyzed mortality throughout the pandemic, regardless of which mutation was prevalent at a particular time.

**Fig. 1:**
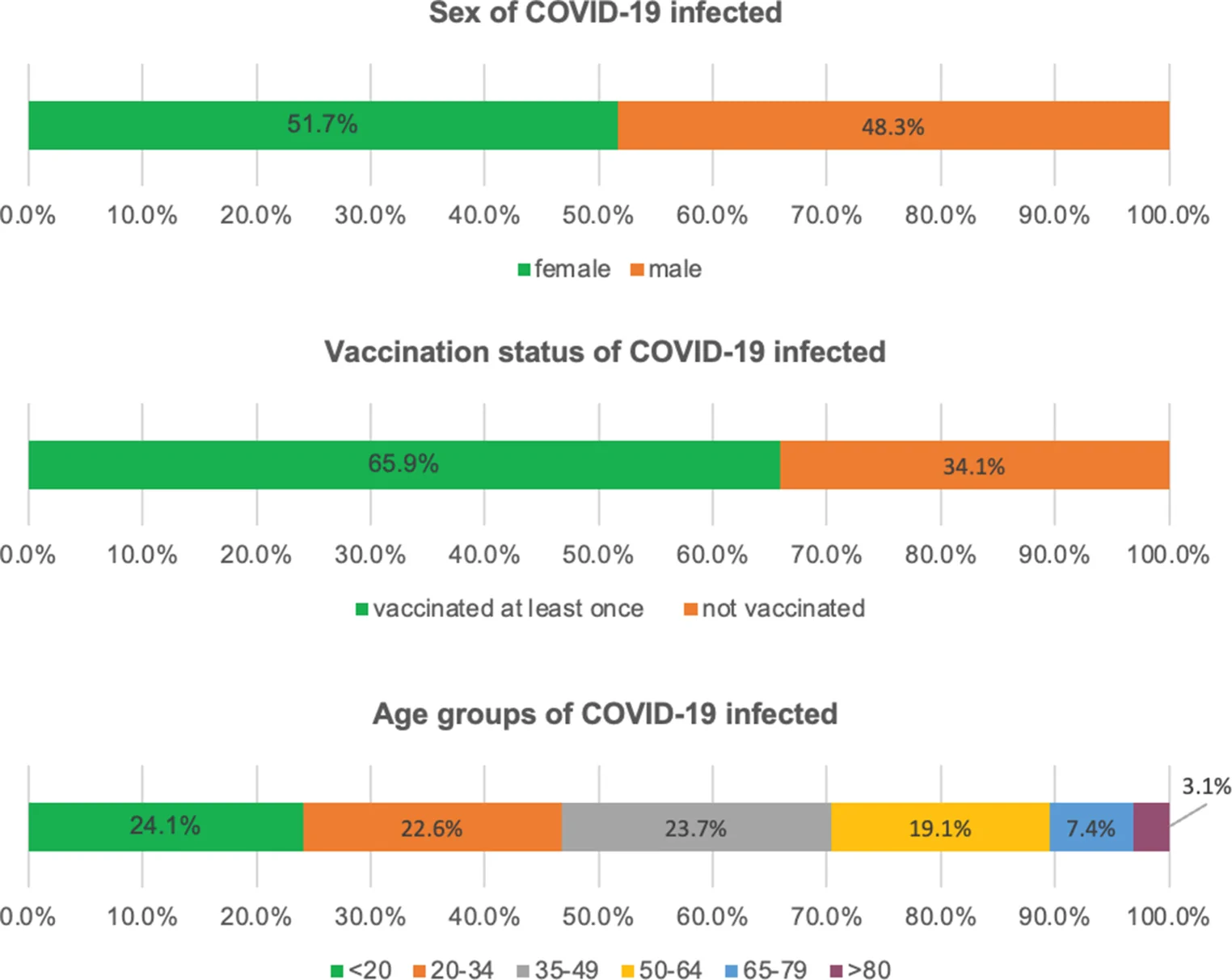
Sex, vaccination status and age (in years) groups of Austrian patients with COVID-19 in percent

After duplicate data entry was removed via MS SQL [15], 4,591,739 confirmed COVID-19 cases, including reinfections, and 20,123 COVID-19-related deaths in Austria were identified. Among the infected individuals, 51.7% were women, 48.3% were men, and 65.9% had been vaccinated at least once. The age distribution of the patients included 34.1% under 20 years, 22.6% between 20–34 years, 23.7% between 35–49 years, 19.1% between 50–64 years, 7.4% between 65–79 years, and 3.1% over 80 years (Fig. 1).

### Statistical analysis

Several models were employed for data analysis. We used a linear mixed model to examine whether individuals died from COVID-19 (binary outcome: 0 = alive, 1 = dead). The model regressed death status on vaccination status (coded 0 = never vaccinated, 1 = vaccinated), age group (coded by the specified age ranges), and sex (coded 1 = male, 2 = female), with male and unvaccinated individuals serving as reference categories. The district of residence was included as a random factor to account for regional variability. We further calculated several interactions with models integrating the time since the beginning of the vaccination from 2021-01-25 until the end of the available data on 2022-07-14, in consecutive months from 1–18, to investigate whether the effects of the fixed factors (vaccination, age and sex) changed over time. We created three models: (i) a model with an interaction between vaccination status and consecutive month, (ii) a model with an interaction between age and consecutive month and (iii) a model with an interaction between sex and consecutive month. In each model, we controlled for age and sex on the basis of a binomial error structure. To estimate the effect sizes, we calculated the odds ratio for the fixed factors.

We also carried out a sensitivity analysis by varying key predictors (vaccination status, age, sex, progressing month) in a reduced prediction dataset and comparing the resulting changes in the model’s predicted probabilities and found that the model reacts plausibly to changes in inputs (lower predicted risk for the vaccinated, higher for men). The model behaves consistently and can be considered robust in its predictions (data not shown).

For the random factors the district Tulln was chosen as the reference category because of its proximity to the average case fatality ratio of all districts. All models include all 116 districts of Austria, which vary geographically and demographically [16].

Additional linear models, which are based on a Gaussian error structure and aggregated data for each district, were computed with the infection ratio and case fatality ratio by district as dependent variables, controlling for the proportion of the population in a district that is over 65 years (ranging from 13.9–26.2%, depending on the district) [17] All variables had been scaled before included in the models (subtracting the mean and dividing by the standard deviation). Only models showing statistically significant associations are presented in the results section.

All analyses were performed in R 4.1.2 (R Foundation for Statistical Computing, Vienna, Austria) [18].

As we examined associations between multiple district-level sociodemographic characteristics and thus involving multiple statistical tests, the risk of chance findings cannot be excluded. Therefore, results from aggregated models should be interpreted as exploratory rather than confirmatory. Furthermore, it is important to emphasize that all district-level analyses are correlational in nature. While several associations align with existing literature, causal interpretations cannot be drawn from these models. Observed associations may reflect unmeasured confounding, demographic composition, or contextual factors rather than direct causal effects. Significance level abbreviations: *p < 0.05; **p < 0.01; ***p < 0.001

## Results

### Main models

The main linear mixed model was regressed on ever being vaccinated, age sex and the number of consecutive months, with district as the random factor, based on a binomial error structure. The results show a significant negative correlation between vaccination status and the risk of mortality from COVID-19. The odds ratio of 0.155 also indicates a significant reduction in mortality due to vaccination. In contrast, age was found to be highly positively associated with mortality risk, as indicated by the odds ratio of 7.4. Being female was found to significantly lower the risk of dying from COVD-19 (negative association, odds ratio of 0.54), as it holds true for progressing in time (month index, odds ratio of 0.816, see Table 1). This indicates that the longer the pandemic progressed, the less fatal COVD-19 became.

**Table 1:** Main linear mixed model: being alive or having died, regressing on being vaccinated, age, sex and progressing time (month index), with Austrian district as a random effect on the basis of a binomial error structure. The table shows the estimates, standard error,* t*-values, *p*-values and odds ratio. Note that in the case of age, the intervals are unequal

	Value	Std. error	*t*-value	*p*-value	Odds ratio
Vaccinated (ref. not vaccinated)	−1.866	0.033	−56.658	*P* < 0.0001	0.155
Age	2.002	0.020	102.505	*P* < 0.0001	7.402
Women (ref. men)	−0.616	0.033	−18.885	*P* < 0.0001	0.540
Month index	−0.203	0.003	−64.995	*P* < 0.0001	0.816
Random effects	Intercept	Residuals	–	–	–
Std. deviation	0.256	1.605	–	–	–

The interaction between vaccination status and progressing month indicates that the strong protective effect of vaccination against COVID-19 mortality decreased gradually over time. The main vaccination effect reflects a substantial reduction in mortality risk at the reference time point (month 0), while the positive interaction term shows that this protective effect became less pronounced with each additional month. Importantly, this interaction represents a per month attenuation of vaccine effectiveness rather than a cumulative loss across the entire observation period. Even at the end of the study period, vaccination remained strongly associated with a markedly reduced risk of death (see Table 2).

**Table 2:** Linear mixed model: being alive or having died, regressing of on being vaccinated interacting with the progressing time (month index), age, sex, progressing time, with Austrian district as a random effect, on basis of binomial error structure; including the interaction of being vaccinated and the progressing time (moth index), Estimates, Std. error, *t*-values, *p*-values, odds ratio

	Value	Std. Error	*t*-value	*p*-value	Odds ratio
Vaccinated (ref. not vaccinated)	−5.058	0.127	−39.921	*P* < 0.0001	0.006
Age	2.009	0.018	112.046	*P* < 0.0001	7.455
Women (ref. men)	−0.596	0.030	−20.148	*P* < 0.0001	0.551
Month index	−0.287	0.004	−66.936	*P* < 0.0001	0.751
Vaccinated × month index	0.155	0.006	26.218	*P* < 0.0001	1.168
Random effects	Intercept	Residuals	–	–	–
Std. deviation	0.0021	1.325	–	–	–

Age was strongly positively associated with COVID-19 mortality, whereas progressing time was associated with an overall decline in mortality risk. The positive interaction between age and progressing month indicates that this temporal decline in mortality was less pronounced for older age groups. Thus, while mortality risk continued to decrease over time across all age groups, the rate of decline was slower among older individuals. These findings reflect effect modification by age rather than an increase in mortality risk among older age groups as the pandemic progressed (see Table 3).

**Table 3:** Linear mixed model: being alive or having died, regressing on age interacting with progressing time (month index), sex, age, with Austrian district as a random effect, on basis of binomial error structure. Estimates, Std. Error, *t*-values, *p*-values, odds

	Value	Std. Error	*t*-value	*p*-value	Odds ratio
Vaccinated (ref. not vaccinated)	−1.877	0.047	−39.652	*P* < 0.0001	0.153
Age	1.386	0.107	12.973	*P* < 0.0001	4.001
Month index	−0.368	0.029	−12.853	*P* < 0.0001	0.692
Women (ref. men)	−0.607	0.047	−12.933	*P* < 0.0001	0.545
Age × month index	0.031	0.005	5.835	*P* < 0.0001	1.031
Random effects	Intercept	Residuals	–	–	–
Std. deviation	0.0035	2.149	–	–	–

Female sex was associated with a lower risk of COVID-19 mortality at the reference time point. The positive interaction between sex and progressing month indicates that the mortality advantage observed among women diminished slightly over time. Nevertheless, overall mortality risk declined for both sexes throughout the observation period, and women continued to exhibit a lower risk of death compared to men. As with age, this interaction reflects a reduced rate of mortality decline rather than a reversal of sex-specific risk patterns. (see Table 4).

**Table 4:** Linear mixed model: being alive or having died, regressing on sex interacting with progressing time (month index), age, sex, progressing time, with Austrian district as a random effect, on basis of binomial error structure. Estimates, std. error, *t*-values, *p*-values, odds ratio.

	Value	Std. Error	*t*-value	*p*-value	Odds ratio
Vaccinated (ref. not vaccinated)	−1.864	0.032	−57.643	*P* < 0.0001	0.155
Age	2.005	0.019	104.271	*P* < 0.0001	7.425
Women (ref. men)	−1.075	0.130	−8.257	*P* < 0.0001	0.341
Month index	−0.213	0.004	−51.024	*P* < 0.0001	0.808
Sex × month index	0.022	0.006	3.640	*P* < 0.0001	1.023
Random effects	Intercept	Residuals	–	–	–
Std. deviation	0.0023	1.460	–	–	–

**Fig. 2:**
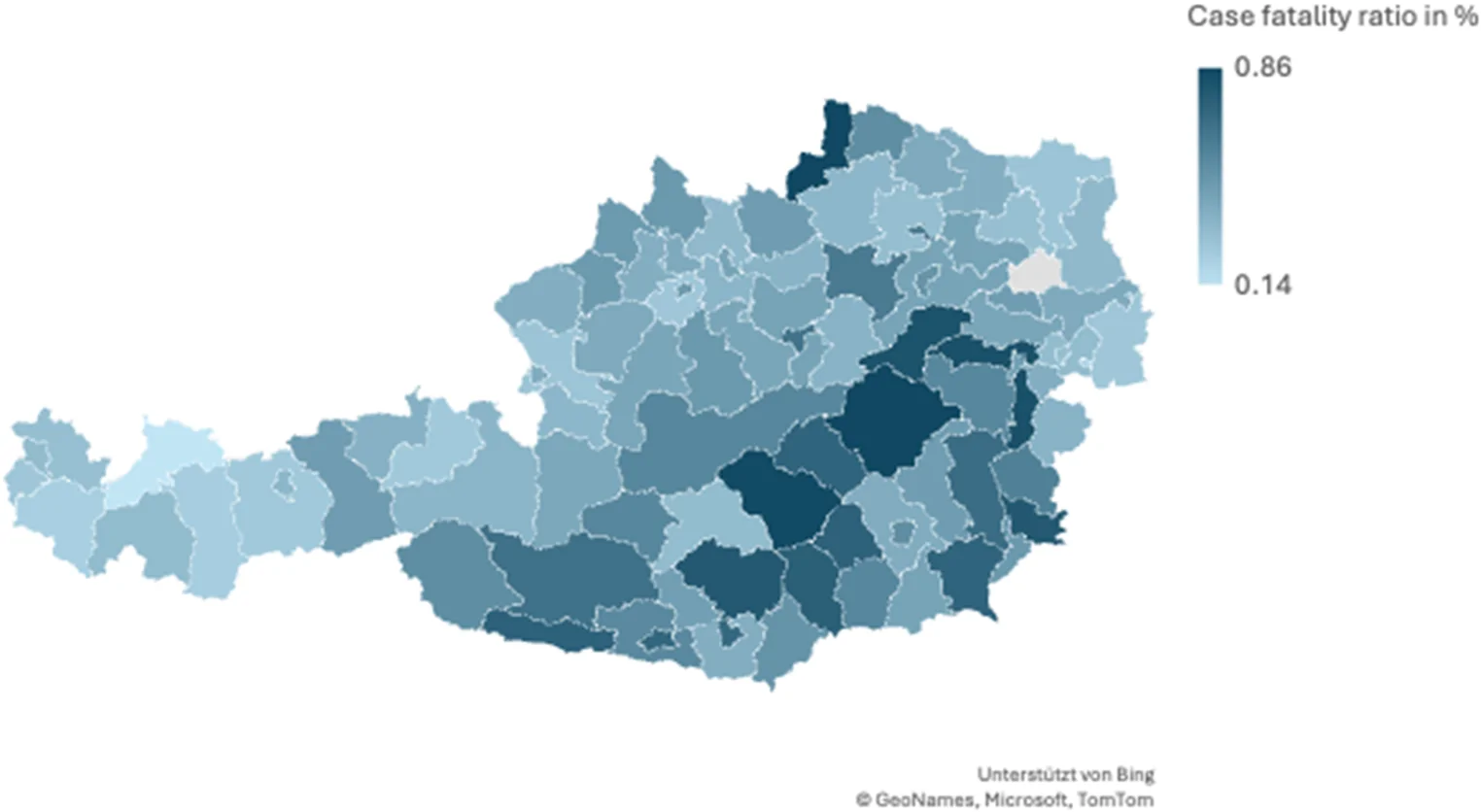
Case fatality ratio in % in all Austrian districts (without Vienna)

The estimates and significances for the fixed factors remained constant when districts were incorporated as additional fixed factors within the primary linear model. Consequently, there was no necessity to re-run the results for the fixed factors; however, a number of significant discrepancies were identified in the case fatality rates among the Austrian districts in comparison to the reference district Tulln (see Tables 5 and 6 in the Appendix). The mean fatality ratio is 0.44% but this varies from 0.14% (District Reutte in Tyrol) to 0.86% (District Gmünd in Lower Austria), depending on the district (see Fig. 2 and Table 6 in the Appendix). This phenomenon also gives rise to disparities in the mortality rate across districts, which ranges from 0.07% (District Reutte in Tyrol) to 0.45% (District Lilienfeld in Lower Austria) (see Table 6 in the Appendix). Further the standard deviation of the intercept of the random factors indicates that Austrian districts contribute only to a lesser extent to the risk of dying of COVID-19, particularly in the interaction models.

### Household size and infection ratio

Controlling for the proportion of people over 65 years we found that the household size was significantly positively associated with the risk of ever being infected with COVID-19 (beta 0.34**). Household size explains about 7% (R2 = 0.07) of the variance of the model (total variance, explained by the model = 27.25%, R2 = 0.2725).

### Tertiary education and case fatality ratio

Again, controlling population age (percentage of individuals over 65), we also find that the proportion of tertiary educated individuals in a district is significantly negatively associated with ever being infected and later dying from COVID-19. (beta = −0.1523**, variance explained = 4.3%, R^2^ = 0.043, total variance explained by the model = 15.8%, R2 = 0.1581) This means that the larger the proportion of tertiary educated individuals in a district, the lower the COVID-19 case fatality ratio.

### Non-Austrian citizenship and case fatality ratio

Controlling for the proportion of people over 65 years in a district, we found that a similar negative association also holds for the association between the share of people with non-Austrian citizenship in a district and the case fatality rate for COVID-19 in a district; beta = −0.3001**, R^2^ = 0.0054. Overall variance explained by the model = 20.86%, R^2^ = 0.2086. The greater the share of people with non-Austrian citizenship in a district, the lower the share of people who died from COVID-19 after being infected; however, the proportion of individuals with non-Austrian citizenship accounts for only approximately 0.5% of the model’s variance.

### Confounding effects of age (population over 65 years old)

Comparisons of models with and without adjustment for the proportion of residents aged over 65 years of age showed that the association between household size and infection ratio persisted after age adjustment. In contrast, the association between tertiary education and COVID-19 mortality was attenuated after controlling for age structure, while the association with non-Austrian citizenship emerged only after age adjustment, indicating strong confounding by district age composition.

## Discussion

In our study, we analyzed the Austrian COVID-19 dataset, which was provided by the Austrian National Public Health Institute. A total of 4,591,739 confirmed COVID-19 cases were recorded up to 14 July 2022. Among the confirmed cases, 20,123 were fatal. Among all those infected, 34.1% had never been vaccinated against COVID-19 (see Fig. 1).

Research has shown that several factors influence COVID-19 mortality, including age [7], sex [19], immigration status [8], household size [6], education level [9] and vaccination status [10]. A difference in mortality can even be observed at the district level [5].

Similar to existing literature [5–10], we also found factors that influence COVID-19 mortality in Austria. Age was found to be highly positively associated with mortality risk: the older the person, the higher the risk.

Being vaccinated was significantly negatively associated with the risk of dying from COVID-19 (odds ratio 0.155). Being female was also associated with a significantly lower risk of dying from COVID-19 (odds ratio 0.540).

As the pandemic progressed, we found that it became less fatal (odds ratio 0.816). Hence, the risk of dying in July 2022 compared to January 2021 was reduced by 0.184. This may be due to increased immunity due to increased vaccination coverage [20] or improved therapeutic options such as nirmatrelvir and ritonavir tablets [21].

The interaction analyses indicate that the associations between vaccination status, age, sex, and COVID-19 mortality were not constant over time but changed in magnitude as the pandemic progressed. In all interaction models, overall mortality risk declined over time; however, the rate of decline differed across subgroups. The protective effect of vaccination remained substantial throughout the observation period but attenuated gradually, consistent with waning immunity or changes in exposure and treatment over time. Similarly, although older age was strongly associated with higher mortality risk, the temporal decrease in mortality was slower among older individuals, indicating effect modification rather than an increase in risk.

A comparable pattern was observed for sex, with women maintaining a mortality advantage over men despite a modest reduction in the magnitude of this advantage over time. Importantly, none of the interaction effects suggest a reversal of risk; instead, they reflect differential rates of decline across subpopulations as the pandemic evolved.

Overall, as indicated by the low standard deviation of the intercept, Austrian districts contribute only to a lesser extent to the risk of dying of COVID-19, particularly in the interaction models. We also found several significant differences in the case fatality ratio and mortality ratio among the Austrian districts. The mean case fatality ratio is 0.44% but ranges from 0.14% (District Reutte in Tyrol) to 0.86% (District Gmünd in Lower Austria), depending on the district (see Table 6). This also led to differences in the mortality ratio among the districts, which ranged from 0.07% (District Reutte in Tyrol) to 0.45% (District Lilienfeld in Lower Austria).

Several factors influence incidence and mortality in different districts. A district’s mean household size is significantly positively associated with the risk of ever being infected with COVID-19. The larger the household, the greater the risk of infection; however, we only have data on household size in terms of number of people and not in terms of square metres available per person. Therefore, it is not possible to say whether these larger households are overcrowded or not. Historically, overcrowding has been linked to increased transmission of respiratory infections, highlighting the importance of social and environmental factors in disease spread [19]. That this is also the case with COVID-19 is supported by multiple studies demonstrating that household crowding not only increases infection risk but also severe COVID-19 outcomes requiring hospitalization [22], with UK Biobank data showing significantly higher odds of severe COVID-19 among individuals in households of three or more compared to two-person households [23].

The proportion of tertiary educated individuals in a district is significantly negatively associated with ever being infected and later dying from COVID-19. The larger the proportion of tertiary educated individuals in a district, the lower the COVID-19 case fatality ratio. Education is a key structural determinant influencing health outcomes through pathways such as health literacy, socioeconomic status, and access to healthcare. The inverse association between district-level tertiary education attainment and COVID-19 case fatality ratio aligns with foundational models of social determinants of health, particularly the World Health Organization’s framework positioning education as a structural driver of health inequities through its influence on economic opportunities, health literacy, and access to protective resources [24]. Empirical evidence supports this conceptualization, showing that education level is consistently associated with reduced risk of COVID-19 infection and mortality, partly due to better understanding of transmission prevention, higher health literacy, and greater economic stability enabling adherence to public health measures [25].

Another negative association was found between the share of people with non-Austrian citizenship and the case fatality ratio for COVID-19 in a district. The greater the share of people with non-Austrian citizenship in a district, the lower the share of people who die from COVID-19 after being infected; however, this result should be interpreted with caution. As people without Austrian citizenship tend to be younger, this influences mortality. The fact that younger people and non-Austrian citizens are connected can be seen in Vienna, which is the federal province with the highest share of the population with non-Austrian citizenship (31.1%) and the lowest share of people who are over 65 years old (16.6%) [17].

This assumption is supported by the finding that including vs. excluding the proportion of the populations aged over 65 years in the models demonstrate that district age structure substantially influences aggregated associations. Whereas the household-size association appears robust to age adjustment, the results for education and non-Austrian citizenship are largely driven by demographic composition. Accordingly, these district-level findings should be interpreted as correlational and exploratory rather than causal; however, although causal links are possible, the data are only correlational, so we cannot make any final claims about causality.

### Limitations

A total of 4,591,739 confirmed COVID-19 cases were recorded up to 14 July 2022 in Austria; however, these cases do not represent individual patients. Each infection was recorded as a case, regardless of whether it was a first-time infection or a reinfection. This means that we cannot distinguish between cases that are initial infections and cases that are reinfections.

Furthermore, the data set only represents infections in individuals who have been tested. Infections that have not been officially reported or have not even been diagnosed through a test are not included.

Another limitation is that we could not obtain data on the precise age structure of the districts, meaning we were unable to correct our model for this. The absence of detailed, age-stratified data at the district level introduces a potential confounding factor that may influence the observed relationship between non-Austrian citizenship share and COVID-19 mortality, as demographic heterogeneity, particularly age distribution, plays a critical role in COVID-19 outcomes. Although the models adjust for the proportion of individuals over 65 years old, this binary categorization cannot fully capture the nuanced variations in age structure across districts, such as the proportion of middle-aged or younger populations, which could significantly impact mortality ratio. Recognizing this constraint underscores the importance of obtaining more granular age data in future research to disentangle the intertwined effects of age, citizenship, and other social determinants on COVID-19 mortality.

We also performed a sensitivity analysis, varying key predictors (vaccination status, age, sex, month of progression) within a reduced prediction dataset, and compared the resulting changes in the model’s predicted probabilities. We found that the model reacted plausibly to changes in the inputs (lower predicted risk for the vaccinated, higher risk for men).

## Conclusion

This study provides insights into the multifactorial determinants of COVID-19 mortality across Austrian political districts by applying statistical modelling to a comprehensive dataset. We found that old age is associated with a high risk of dying from COVID-19. In contrast, being vaccinated lowers the risk of dying from COVID-19. Being female is also linked with a lower risk of death from the disease, which became less fatal as the pandemic progressed; however, the risk of death did increase slightly for older women (and men). The effectiveness of the vaccination decreased as the pandemic progressed. Differences in Austrian districts contribute only to a lesser extent to the difference in death risk.

District factors also affect incidence and mortality: higher household sizes are correlated with higher infection ratio, whereas districts with more individuals with tertiary education have lower infection and mortality ratio. Districts with more residents without Austrian citizenship have lower case fatality ratio, likely because of younger populations. These findings highlight the critical role of vaccination in reducing COVID-19 mortality. Future studies should further explore how socioeconomic, healthcare system, and community-level interventions can reduce excess mortality in high-risk districts; however, district age structure (proportion of older individuals) substantially influences these associations.

### Policy recommendations

In the event of another pandemic or a similar public health emergency, policy makers must utilize the knowledge gained to implement appropriate measures from the very beginning. Notably, age and vaccination collectively explain more than 85% of mortality variance, underscoring their primacy relative to regional and demographic factors. These results reinforce the established understanding that population structure and immunization rates are central drivers in pandemic outcomes. The clear link between higher vaccination coverage and a lower risk of death means it is essential to invest financial resources in vaccination campaigns, particularly for older populations. Recent country-level studies and meta-analyses confirm that vaccinated individuals are significantly less likely to die of COVID-19 than nonvaccinated individuals, and that increased vaccination coverage reduces excess mortality even when adjusted for confounding factors such as life expectancy or comorbidities [26, 27]. Vaccination is also associated with lower incidence rate, severeness and hospitalization [11, 28]. Furthermore, regional disparities in mortality necessitate targeted policy recommendations. District-level observations and resource allocation must be refined in order to address local vulnerabilities, such as disparities in educational attainment.

## Appendix

**Table 5:** Main linear model: Differences in the case fatality ratio among Austrian districts, with Tulln as the reference district (estimate, std. error, z‑value and *p*-values), linear model on the basis of a binomial error structure

District	Estimate	Std. Error	z‑value	*p*-value
Reference: Tulln	0.0000	0.0000	0.0000	0.0000
Eisenstadt	−0.2730	0.2416	−1.1300	0.2585
Rust	−0.6901	0.7399	−0.9330	0.3510
Eisenstadt-Umgebung	−0.1113	0.1512	−0.7360	0.4616
Güssing	0.2582	0.1400	1.8450	0.0650
Jennersdorf	−0.2359	0.1968	−1.1990	0.2307
Mattersburg	−0.0609	0.1498	−0.4070	0.6842
Neusiedl am See	−0.3159	0.1419	−2.2270	0.0260
Oberpullendorf	−0.1607	0.1564	−1.0280	0.3042
Oberwart	0.3032	0.1209	2.5070	0.0122
Klagenfurt am Wörthersee	0.1490	0.1005	1.4820	0.1384
Villach	0.0191	0.1117	0.1710	0.8643
Hermagor	−0.2726	0.1621	−1.6820	0.0926
Klagenfurt-Land	−0.5622	0.1315	−4.2750	0.0000
St. Veit an der Glan	0.2459	0.1130	2.1760	0.0296
Spittal an der Drau	−0.2734	0.1061	−2.5770	0.0100
Villach-Land	−0.2746	0.1156	−2.3760	0.0175
Völkermarkt	−0.3215	0.1337	−2.4050	0.0162
Wolfsberg	0.1353	0.1144	1.1830	0.2368
Feldkirchen	−0.5483	0.1574	−3.4830	0.0005
Krems an der Donau	0.1768	0.1548	1.1420	0.2534
St. Pölten	−0.0152	0.1221	−0.1240	0.9010
Waidhofen an der Ybbs	−0.2136	0.1915	−1.1150	0.2647
Wiener Neustadt	0.7250	0.1149	6.3080	0.0000
Amstetten	−0.2372	0.0997	−2.3790	0.0174
Baden	0.0328	0.0990	0.3310	0.7405
Bruck an der Leitha	0.1673	0.1081	1.5470	0.1217
Gänserndorf	−0.0438	0.1146	−0.3820	0.7025
Gmünd	0.3431	0.1204	2.8500	0.0044
Hollabrunn	−0.1859	0.1399	−1.3290	0.1839
Horn	−0.2955	0.1622	−1.8210	0.0685
Korneuburg	−0.0765	0.1215	−0.6290	0.5293
Krems	−0.5500	0.1362	−4.0380	0.0001
Lilienfeld	0.2209	0.1334	1.6560	0.0978
Melk	0.1520	0.1023	1.4870	0.1371
Mistelbach	−0.2660	0.1324	−2.0080	0.0446
Mödling	0.0789	0.1031	0.7650	0.4441
Neunkirchen	0.0031	0.1049	0.0290	0.9766
St. Pölten	−0.0633	0.1000	−0.6330	0.5264
Scheibbs	−0.5099	0.1377	−3.7030	0.0002
Waidhofen an der Thaya	−0.2640	0.1572	−1.6800	0.0930
Wiener Neustadt	−0.1119	0.1121	−0.9980	0.3183
Zwettl	−0.6745	0.1423	−4.7390	0.0000
Linz	−0.0315	0.0942	−0.3340	0.7382
Steyr	−0.5091	0.1558	−3.2690	0.0011
Wels	0.0057	0.1190	0.0480	0.9619
Braunau	−0.4171	0.1042	−4.0050	0.0001
Eferding	−0.1572	0.1532	−1.0260	0.3049
Freistadt	−0.1844	0.1137	−1.6210	0.1049
Gmunden	−0.2412	0.1052	−2.2930	0.0219
Grieskirchen	−0.3597	0.1195	−3.0090	0.0026
Kirchdorf	−0.1872	0.1196	−1.5650	0.1176
Linz-Land	−0.1881	0.1013	−1.8570	0.0634
Perg	−0.2534	0.1188	−2.1330	0.0329
Ried	−0.1367	0.1159	−1.1800	0.2381
Rohrbach	−0.1069	0.1178	−0.9080	0.3641
Schärding	−0.1634	0.1191	−1.3720	0.1700
Steyr-Land	−0.2451	0.1200	−2.0430	0.0410
Urfahr-Umgebung	−0.3101	0.1153	−2.6900	0.0071
Vöcklabruck	−0.2314	0.0981	−2.3590	0.0183
Wels-Land	−0.5942	0.1271	−4.6740	0.0000
Salzburg	−0.2938	0.0980	−2.9990	0.0027
Hallein	−0.4476	0.1221	−3.6660	0.0002
Salzburg-Umgebung	−0.5796	0.1008	−5.7510	0.0000
St. Johann im Pongau	−0.2898	0.1099	−2.6380	0.0083
Tamsweg	−0.0931	0.1601	−0.5820	0.5609
Zell am See	−0.3572	0.1142	−3.1270	0.0018
Graz	0.2841	0.0879	3.2330	0.0012
Deutschlandsberg	−0.1536	0.1158	−1.3270	0.1845
Graz-Umgebung	−0.2252	0.1000	−2.2520	0.0243
Leibnitz	−0.1476	0.1124	−1.3140	0.1889
Leoben	0.1542	0.1155	1.3360	0.1816
Liezen	−0.2286	0.1083	−2.1110	0.0348
Murau	−0.7530	0.1900	−3.9640	0.0001
Voitsberg	0.0468	0.1176	0.3980	0.6906
Weiz	−0.1395	0.1078	−1.2940	0.1957
Murtal	0.3108	0.1040	2.9890	0.0028
Bruck-Mürzzuschlag	0.2409	0.0988	2.4390	0.0147
Hartberg-Fürstenfeld	0.1218	0.1012	1.2030	0.2290
Südoststeiermark	0.1032	0.1017	1.0150	0.3101
Innsbruck (I)	−0.3225	0.1050	−3.0710	0.0021
Imst	−0.7754	0.1389	−5.5830	0.0000
Innsbruck-Land	−0.5711	0.1016	−5.6220	0.0000
Kitzbühel	−0.8829	0.1361	−6.4860	0.0000
Kufstein	−0.3815	0.1052	−3.6260	0.0003
Landeck	−0.6122	0.1410	−4.3410	0.0000
Lienz	−0.3899	0.1207	−3.2300	0.0012
Reutte	−1.3608	0.2267	−6.0010	0.0000
Schwaz	−0.1420	0.1092	−1.3010	0.1932
Bludenz	−0.8237	0.1384	−5.9520	0.0000
Bregenz	−0.4095	0.1061	−3.8600	0.0001
Dornbirn	−0.2976	0.1159	−2.5660	0.0103
Feldkirch	−0.4541	0.1135	−4.0010	0.0001
Wien 1., Innere Stadt	−1.5444	0.2265	−6.8170	0.0000
Wien 2., Leopoldstadt	0.2166	0.1032	2.0990	0.0358
Wien 3., Landstraße	−0.5006	0.1208	−4.1450	0.0000
Wien 4., Wieden	−0.2709	0.1626	−1.6660	0.0958
Wien 5., Margareten	0.2981	0.1296	2.3000	0.0214
Wien 6., Mariahilf	0.1042	0.1615	0.6450	0.5187
Wien 7., Neubau	−0.2840	0.1970	−1.4410	0.1495
Wien 8., Josefstadt	−0.5184	0.2337	−2.2180	0.0266
Wien 9., Alsergrund	−0.1252	0.1586	−0.7900	0.4297
Wien 10., Favoriten	0.2384	0.0928	2.5680	0.0102
Wien 11., Simmering	0.2535	0.1102	2.3010	0.0214
Wien 12., Meidling	0.2669	0.1069	2.4980	0.0125
Wien 13., Hietzing	0.1817	0.1181	1.5380	0.1240
Wien 14., Penzing	−0.1293	0.1080	−1.1980	0.2310
Wien 15., Rudolfsheim-Fünfhaus	0.0880	0.1224	0.7180	0.4725
Wien 16., Ottakring	0.2792	0.1073	2.6010	0.0093
Wien 17., Hernals	0.1503	0.1342	1.1200	0.2627
Wien 18., Währing	0.3396	0.1224	2.7740	0.0055
Wien 19., Döbling	0.0613	0.1078	0.5680	0.5700
Wien 20., Brigittenau	0.3043	0.1107	2.7490	0.0060
Wien 21., Floridsdorf	0.0806	0.1002	0.8050	0.4209
Wien 22., Donaustadt	−0.0308	0.0996	−0.3090	0.7570
Wien 23., Liesing	0.2379	0.1034	2.3010	0.0214

**Table 6:** Main linear model: Austria’s districts with their number of inhabitants, infection ratio and case fatality ratio

Federal province	District	Inhabitants	Infection ratio in %	Case fatality ratio in %	Mortality ratio in %
Burgenland	Eisenstadt	14,895	48.36	0.32	0.15
	Rust	2000	45.4	0.22	0.1
	Eisenstadt-Umgebung	43,861	49.3	0.33	0.16
	Güssing	25,770	47.96	0.78	0.37
	Jennersdorf	17,109	45.6	0.49	0.22
	Mattersburg	40,316	47.65	0.39	0.18
	Neusiedl am See	60,397	49.36	0.28	0.14
	Oberpullendorf	37,453	46.77	0.37	0.17
	Oberwart	54,209	46.5	0.6	0.28
Carinthia	Klagenfurt am Wörthersee	101,765	43.83	0.71	0.31
	Villach	63,236	43.56	0.72	0.31
	Hermagor	18,052	47.04	0.74	0.35
	Klagenfurt-Land	60,503	45.41	0.39	0.18
	St. Veit an der Glan	53,880	46.45	0.78	0.36
	Spittal an der Drau	75,628	47.11	0.67	0.32
	Villach-Land	64,920	45.87	0.56	0.26
	Völkermarkt	41,834	49.52	0.51	0.25
	Wolfsberg	52,488	46.37	0.75	0.35
	Feldkirchen	29,783	46.44	0.46	0.21
Lower Austria	Krems an der Donau (Stadt)	24,837	46.54	0.63	0.29
	St. Pölten (Stadt)	55,878	50	0.51	0.25
	Waidhofen an der Ybbs	11,134	56.92	0.66	0.38
	Wiener Neustadt (Stadt)	46,456	51.54	0.82	0.42
	Amstetten	116,592	60.33	0.44	0.26
	Baden	147,113	50.03	0.43	0.22
	Bruck an der Leitha	105,507	49.83	0.41	0.2
	Gänserndorf	105,824	47.1	0.34	0.16
	Gmünd	36,275	48.99	0.86	0.42
	Hollabrunn	51,332	45.71	0.39	0.18
	Horn	30,838	47.27	0.41	0.19
	Korneuburg	91,777	47.16	0.31	0.15
	Krems (Land)	56,559	51.79	0.32	0.17
	Lilienfeld	25,474	55.21	0.81	0.45
	Melk	78,281	57.78	0.63	0.36
	Mistelbach	75,655	47.28	0.29	0.14
	Mödling	119,240	47.12	0.47	0.22
	Neunkirchen	86,323	50.17	0.57	0.29
	St. Pölten (Land)	132,064	52.19	0.44	0.23
	Scheibbs	41,567	60.6	0.37	0.22
	Tulln	105,762	47	0.44	0.21
	Waidhofen an der Thaya	25,531	48.87	0.54	0.26
	Wiener Neustadt (Land)	79,033	51.18	0.46	0.24
	Zwettl	41,827	56.46	0.35	0.2
Upper Austria	Linz	206,537	46.18	0.44	0.2
	Steyr	37,952	46.91	0.37	0.17
	Wels	62,654	50.7	0.49	0.25
	Braunau	106,492	58.67	0.39	0.23
	Eferding	33,368	53.04	0.41	0.22
	Freistadt	66,922	56.99	0.47	0.27
	Gmunden	102,102	53.95	0.43	0.23
	Grieskirchen	65,137	57.4	0.39	0.22
	Kirchdorf	57,163	53.55	0.49	0.26
	Linz-Land	152,391	50.83	0.36	0.18
	Perg	69,241	57.22	0.37	0.21
	Ried	61,850	57.36	0.48	0.27
	Rohrbach	56,623	57.12	0.49	0.28
	Schärding	57,438	54.97	0.48	0.26
	Steyr-Land	60,936	54.34	0.43	0.24
	Urfahr-Umgebung	86,235	54.71	0.34	0.19
	Vöcklabruck	137,993	57.26	0.41	0.24
	Wels-Land	74,574	55.92	0.28	0.15
Salzburg	Salzburg	155,416	52.4	0.4	0.21
	Hallein	60,992	62.1	0.35	0.22
	Salzburg-Umgebung	154,624	61.23	0.29	0.18
	St. Johann im Pongau	81,392	59.31	0.42	0.25
	Tamsweg	20,118	55.88	0.57	0.32
	Zell am See	88,168	52.87	0.36	0.19
Styria	Graz	291,134	45.63	0.54	0.25
	Deutschlandsberg	60,871	48.03	0.57	0.28
	Graz-Umgebung	157,853	47.54	0.4	0.19
	Leibnitz	85,294	48.49	0.44	0.21
	Leoben	59,151	41.27	0.73	0.3
	Liezen	59,151	63.68	0.57	0.36
	Murau	27,449	43.35	0.33	0.14
	Voitsberg	50,947	44.58	0.74	0.33
	Weiz	90,916	50.47	0.47	0.24
	Murtal	71,356	44.27	0.85	0.38
	Bruck-Mürzzuschlag	98,054	41.73	0.86	0.36
	Hartberg-Fürstenfeld	90,619	48.22	0.69	0.33
	Südoststeiermark	83,841	47.72	0.73	0.35
Tyrol	Innsbruck	131,059	51.23	0.35	0.18
	Imst	60,922	57.16	0.25	0.14
	Innsbruck-Land	181,698	53.48	0.27	0.15
	Kitzbühel	64,676	51.97	0.27	0.14
	Kufstein	111,080	55.25	0.38	0.21
	Landeck	44,346	58.58	0.33	0.19
	Lienz	48,814	55.08	0.54	0.3
	Reutte	33,054	51.7	0.14	0.07
	Schwaz	84,456	52.88	0.47	0.25
	Bludenz	64,368	56.55	0.24	0.14
	Bregenz	135,310	53.16	0.31	0.17
	Dornbirn	90,502	54.51	0.33	0.18
	Feldkirch	109,057	55.42	0.28	0.16
Vienna	Wien 1., Innere Stadt	15,867	67.22	0.23	0.15
	Wien 2., Leopoldstadt	105,237	52.46	0.51	0.27
	Wien 3., Landstraße	93,248	58.68	0.25	0.14
	Wien 4., Wieden	33,075	51.33	0.35	0.18
	Wien 5., Margareten	54,373	50	0.45	0.22
	Wien 6., Mariahilf	31,336	52.33	0.38	0.2
	Wien 7., Neubau	31,683	53.83	0.21	0.11
	Wien 8., Josefstadt	24,365	52.05	0.19	0.1
	Wien 9., Alsergrund	41,812	54.84	0.28	0.15
	Wien 10., Favoriten	210,573	52.22	0.43	0.22
	Wien 11., Simmering	105,022	52.6	0.37	0.2
	Wien 12., Meidling	96,998	50.52	0.49	0.25
	Wien 13., Hietzing	53,903	49.63	0.6	0.3
	Wien 14., Penzing	93,366	54.7	0.42	0.23
	Wien 15., Rudolfsheim-Fünfhaus	76,137	50.62	0.37	0.19
	Wien 16., Ottakring	102,480	50.08	0.44	0.22
	Wien 17., Hernals	56,488	48.72	0.39	0.19
	Wien 18., Währing	51,327	50.88	0.58	0.29
	Wien 19., Döbling	73,861	49.29	0.64	0.31
	Wien 20., Brigittenau	85,264	49.59	0.49	0.24
	Wien 21., Floridsdorf	173,916	51.68	0.33	0.17
	Wien 22., Donaustadt	198,806	54.89	0.28	0.15
	Wien 23., Liesing	111,812	51.96	0.46	0.24
